# Lack of Patient and Public Involvement Reporting in Allergy Prevention Trials: The Example of Complementary Feeding

**DOI:** 10.1111/cea.70343

**Published:** 2026-05-20

**Authors:** Jonas Lander, Helen Elizabeth Smith, Johanna Meyer, Theresa Donhauser, Christian Apfelbacher

**Affiliations:** ^1^ Hannover Medical School Institute for Epidemiology, Social Medicine and Health Systems Research Hannover Germany; ^2^ Keele University, School of Medicine Staffordshire UK; ^3^ Institute of Social Medicine and Health Systems Research, Medical Faculty Otto von Guericke University Magdeburg Magdeburg Germany; ^4^ Lee Kong Chian School of Medicine Nanyang Technological University Singapore Singapore

## Abstract

- PPI may add value to the planning, conduct and translation of findings of allergy prevention trials.
- Reporting of PPI in allergy prevention trials is invisible although clearly advocated for by research funding bodies and others.

To the Editor,

Clinical research is essential to uphold and improve health, but patient representatives have long criticized trials for focusing on clinical outcomes rather than patient priorities even if a gradual shift towards quality of life aspects is now visible [1]. Similar discussions have emerged in allergy research [2].

Patient and public involvement (PPI), an approach in which research is conducted with rather than on or for patients, has been recognized and internationally advocated for over two decades. Respective developments have been identified since the early 2000s [3]. Since the late 2000s, PPI became a more formal part of research systems, guideline development and national research strategies (e.g., [4, 5]), accompanied by increasing empirical and conceptual literature (e.g., [6]). Since mid‐2010, practical tools to help integrate PPI in research practice are widely available, including dedicated guidance, evaluation tools and reporting frameworks (e.g., [7]).

In allergy prevention research, PPI could add value at several points of a trial: co‐setting priorities and outcomes (e.g., symptom relief, sleep, parental anxiety); co‐designing real‐life interventions (e.g., feeding routines); tailoring risk communication (e.g., parents with high risk infants); optimizing recruitment beyond tertiary clinics; and co‐creating post‐trial messages for primary care. However, it is unclear to which degree such efforts have been implemented in allergy research.

We assessed the reporting of PPI in clinical trials on complementary feeding (CF) as an example. CF trials typically examine the timing and composition of infant diet, such as the early introduction of peanut or egg. CF interventions constitute a key domain in early allergy prevention that directly affects parental decision‐making. Therefore, we used CF trials to assess if PPI is visible in a domain where parental practices are central to intervention delivery.

The included trials were derived from an up‐to‐date overview of systematic reviews on early allergy prevention by Kuper et al. [8]. The study systematically identified and synthesized randomized CF trials and thus represents the most recent evidence base. We included all identified trials published since 2000 to examine how the increasing international visibility of PPI is reflected in published CF trials.

We used the main themes of the GRIPP2 reporting checklist—developed in 2011 and revised in 2017 [7]—for PPI in research as an analytical framework to identify whether PPI‐related elements were visible in trial reports. We defined and pre‐tested a structured set of *n* = 10 data extraction categories in relation to: PPI context: general consideration of patient‐public perspectives on research, need for patient‐centred research; PPI concept: importance of PPI concept, related PPI concepts (e.g., shared‐decision making); PPI aim/method: interaction among researchers and study participants, information activities, consultation activities, involvement activities; PPI outcome: overall rating as study with PPI elements; PPI reflection: PPI‐related study limitation (see Figure 1).

**FIGURE 1:**
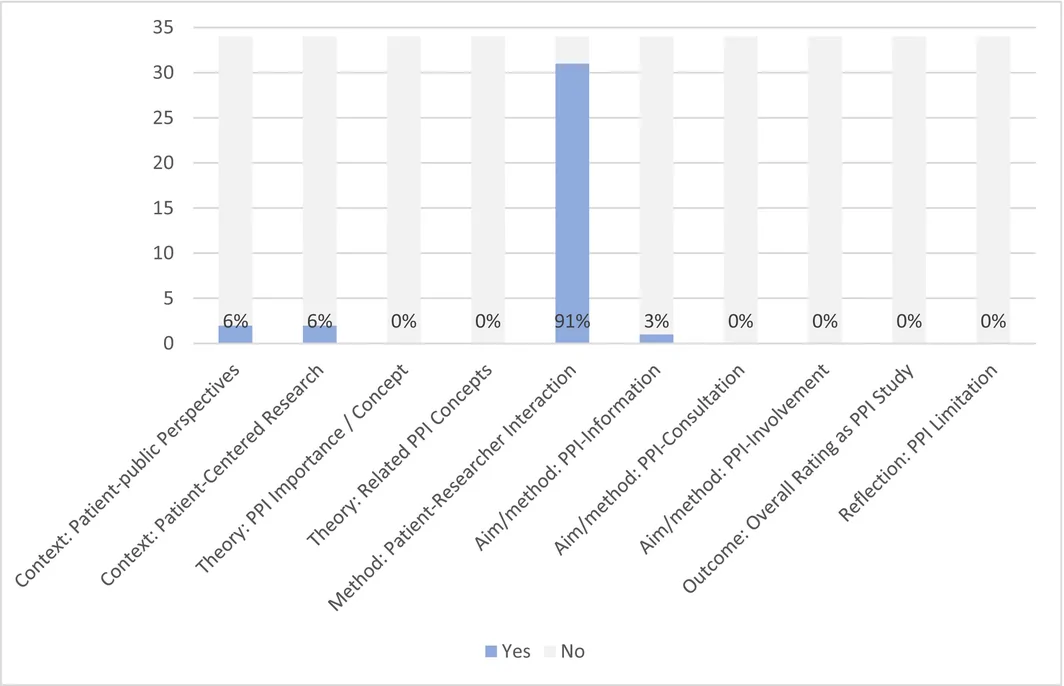
Presence of PPI elements across 34 complementary feeding trials.

Additionally, we collected data on six study characteristics: authors, year of publication, year of study, research objective, study population and funding. Following the pre‐test, two researchers independently checked 25% of trials (*n* = 8), assessed the remaining studies individually and applied consensual coding to resolve unclear cases (54 of 578 total ratings; 9%), reflecting a high level of agreement across predefined, binary extraction categories. Relevant text passages were extracted verbatim, coded, rated ‘yes/no’ and summarized descriptively and narratively.

We included *n* = 34 clinical CF trials, most of which were conducted in the UK (*n* = 8), Australia (*n* = 7), Japan (*n* = 5) and Canada (*n* = 4), all countries which have actively promoted PPI in health research for decades. The majority of trials reported multiple, mixed public‐private funding sources (*n* = 16) such as national research councils, governmental agencies and foundations; *n* = 9 trials provided a single funding source; *n* = 3 reported no funding and *n* = 6 did not provide a funding statement. Most trials were published from 2010 onwards (25/34, 73%). Objectives commonly addressed early introduction of allergenic foods, interventions to reduce allergen exposure or nutritional/developmental outcomes, with allergy prevention often stated as a secondary objective. Targeted populations were mostly high‐risk infants (< 12 months), though some studies recruited healthy infants to assess broader applicability.

Only a few studies described if and how perspectives of parents and families matter for research to contextualize the study. Two (6%) referred to parental values, for example, emotional aspects of weaning, unmet needs in food allergy prevention. Thirteen (38%) mentioned allergy prevalence or burden but without linking these to parents' needs.

None of the 34 trial reports described if PPI was important in general or specific to allergy research, nor referred to related concepts such as patient education, self‐management, empowerment. No study reflected on potential limitations due to the absence of PPI. Two (6%) called for more patient‐centred research, focusing on feasibility and subgroup‐specific effectiveness.

Regarding interaction with study participants, most trial reports (31/34; 91%) described routine clinical assessments, follow‐up visits, questionnaires or symptom diaries completed by parents and less often phone or home follow‐ups. These contacts focused on monitoring adherence, collecting data and ensuring safety rather than involving families as active study contributors.

Only one study (3%) applied a parent information format, using leaflets and posters. None reported consultation formats such as advisory boards, feedback opportunities or active involvement roles, for example, contributing to participant recruitment. Based on information in the scientific literature, we assessed all studies (100%) as not having reported PPI. We cannot exclude the possibility that PPI was conducted but not reported.

Since the included publications often did not report when precisely a trial commenced, and because PPI developed heterogeneously across countries, we could not reliably restrict the sample to trials initiated after country‐specific PPI recommendations. Our findings should be interpreted as a retrospective analysis of PPI visibility in published reports rather than as an assessment of how trials complied with country‐specific PPI policies, which emerged at different times.

In summary, the reporting of PPI is almost completely absent in CF trials, irrespective of funding type or number of funding sources, and this absence persisted in the many more recent trials published after 2020. Since PPI is shaped by structural factors such as research culture, skills and resources, similar reporting gaps may occur across other allergy‐prevention domains.

A recent national survey in Japan examined perspectives of allergy researchers and patient advocates towards PPI [9]: Besides describing what different actors think about PPI, evidence about its actual role in clinical allergy trials is necessary. To complete our retrospective analysis of published reports, future work should investigate barriers to the adoption of PPI practices with trialists directly in other cultural contexts. Importantly, when involving parents with small children in research, family routines need to be explicitly addressed.

Professional allergy societies should take a more active role in promoting PPI: For instance, they could adapt existing PPI guidance to the specific context of allergy research, including minimum standards and practicable formats for trials. They could also promote awareness of PPI with workshops and perhaps even PPI awards. Within each research team there is the potential to collaborate with participating families at trial onset in PPI advisory groups, jointly discussing intervention burdens and risks to refine the intervention and reduce loss to follow up.

## Author Contributions

Study design: J.L., C.A. Data analysis: J.L., J.M., T.D. Interpretation of results: J.L., H.E.S., C.A. Writing of manuscript: J.L. Reviewed and approved manuscript: all authors.

## Funding

The authors have nothing to report.

## Ethics Statement

This study did not involve humans.

## Conflicts of Interest

The authors declare no conflicts of interest.

## Data Availability

The data that support the findings of this study can be obtained from the corresponding author upon request.
